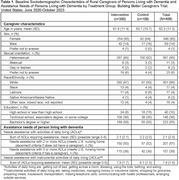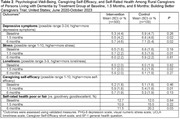# Online, Community‐Based Workshop for Rural Family Caregivers of Persons Living with Dementia in the United States: Results of a National Randomized Controlled Trial

**DOI:** 10.1002/alz.087787

**Published:** 2025-01-09

**Authors:** Veronica Yank, Jasmine Santoyo‐Olsson, Jing Cheng, Kate Lorig, Dolores Gallagher‐Thompson, Catherine Chesla, Leah Karliner, Kenneth E Covinsky, Elizabeth Macias Romo, Maritza Luzanilla

**Affiliations:** ^1^ University of California San Francisco, San Francisco, CA USA; ^2^ Stanford University, Stanford, CA USA

## Abstract

**Background:**

Family caregivers of persons living with dementia (PLWD) in rural areas of the United States (U.S.) are isolated, under‐served, and experience poor health outcomes. We evaluated the impact of an online workshop on the psychological well‐being and health of rural caregivers.

**Methods:**

We conducted a randomized controlled trial of an asynchronous 6‐week online peer‐led small group workshop, Building Better Caregivers, that is designed to increase self‐care and caregiving skills. Caregivers use materials at home whenever they have time, self‐pace their learning, and chat asynchronously with other caregivers through threaded discussion board conversations. Caregivers living in U.S. rural areas, 18+ years old, and giving care ≥ 10 hours a week to a family member or friend living with dementia were randomly assigned in a 3:1 ratio to intervention or attention control (phone calls and caregiver handbook, plus workshop after trial completion). Data were collected at baseline, 1.5 months, and 6 months. Primary outcomes were depressive symptoms and stress. Secondary outcomes were loneliness, caregiving self‐efficacy, and self‐reported health. Longitudinal outcomes were modeled with (generalized) linear mixed effect models and compared with corresponding contrasts between the two groups over time.

**Results:**

409 caregivers participated, and 88% were retained at 6 months. Participants were aged 62.3±11.2 years; 85% identified as female; 90% identified as heterosexual; 86% identified as White; 55% had ≥ bachelor’s degree. (Table 1) Most provided care to a spouse (44%), parent (44%), or other relative (7%), and 76% lived with the PLWD. Among PLWD at baseline, 51% needed assistance with at least two activities of daily living (ADLs), and 39% required assistance with three or more ADLs. Compared to controls at 1.5 months, intervention group participants had lower depression symptoms, stress, and loneliness, and higher caregiving self‐efficacy. (Table 2) At 6 months, intervention group participants had fewer depressive symptoms and were less likely to report poor health than controls. Specifically, intervention group caregivers maintained similar levels of depressive symptoms and self‐rated health throughout the trial whereas controls worsened substantially.

**Conclusions:**

Findings suggest that the Building Better Caregivers online workshop improves the well‐being of rural caregivers who perform high burden care.